# Prevalence of Hyperhomocysteinaemia and Associated Factors among Ethiopian Adult Population in a 2015 National Survey

**DOI:** 10.1155/2020/9210261

**Published:** 2020-01-11

**Authors:** Feyissa Challa, Tigist Getahun, Meron Sileshi, Bikila Nigassie, Zeleke Geto, Genet Ashibire, Terefe Gelibo, Solomon Teferra, Daniel Seifu, Yohannes Sitotaw, Abebe Bekele, Ebba Abate, Kaleab Baye

**Affiliations:** ^1^National References Laboratory for Clinical Chemistry, Ethiopian Public Health Institute, Addis Ababa, Ethiopia; ^2^ICAP-CU, Addis Ababa, Ethiopia; ^3^Department of Psychiatry, School of Medicine, College of Health Sciences, Addis Ababa University, Addis Ababa, Ethiopia; ^4^Department of Biochemistry, School of Medicine, College of Health Sciences, Addis Ababa University, Addis Ababa, Ethiopia; ^5^Ethiopian Biotechnology Institute, Addis Ababa, Ethiopia; ^6^Health System and Reproductive Health Research Directorate, Ethiopian Public Health Institute, Addis Ababa, Ethiopia; ^7^Center for Food Science and Nutrition, College of Natural Sciences, Addis Ababa University, Addis Ababa, Ethiopia

## Abstract

Hyperhomocysteinaemia (HHcy) is an independent risk factor for major cardiovascular diseases, but data on the prevalence and predictors of HHcy in low- and middle-income countries like Ethiopia are scant. The aim of this study was to estimate the prevalence of HHcy and associated risk factors in the Ethiopian adult population. A cross-sectional survey on risks of noncommunicable diseases (NCDs) using the STEPwise approach to surveillance (STEPS) survey was conducted between April and June 2015. A total of 4,175 study participants were surveyed. Serum homocysteine (Hcy) and metabolic profile were determined using Cobas Integra 400 Plus and CardioChek PA analyzer, respectively. Factors associated with HHcy were determined using logistic regression. The mean serum tHcy concentration was 14.6 *μ*mol/L, with 16.4 *μ*mol/L in males and 13.4 *μ*mol/L in females. Overall, 38% had HHcy, with figures in males (49%) higher than females (30%). Increased age, being male, and high blood pressure and/or taking blood pressure medication, as well as low consumption of fruit and/or vegetables, were independent risk factors for HHcy. In conclusion, the prevalence of HHcy among the adult Ethiopian population is alarmingly high. Improving diets through the promotion of fruit and vegetable consumption is needed to reduce the risk of NCDs.

## 1. Introduction

Homocysteine (Hcy), *2-methyl-4-mercapto butyric acid*, is a sulfur-containing amino acid required for normal biosynthesis of the amino acids methionine and cysteine [1]. Under normal conditions, serum Hcy concentration is strictly maintained by a complex pathway (remethylation or transsulfuration) which involves different factors such as folic acid, vitamin B12 (cobalamin), vitamin B6 (pyridoxine), methylenetetrahydrofolate reductase enzyme (MTHFR), methionine synthase, and cystathionine beta-synthase [2, 3]. However, folate and B-vitamin deficiencies [4], lifestyle behaviors (e.g., smoking, physical inactivity, and chronic alcohol consumption) [5], and genetic defects (i.e., polymorphisms in MTHFR) [6] are all associated with hyperhomocysteinaemia (HHcy) (Hcy ≥ 15 *μ*mol/L) [7].

HHcy is an independent risk factor for many chronic diseases including cardiovascular-related diseases such as [8] atherosclerosis [9], hypertension [10], stroke and coronary artery diseases [11, 12], and type 2 diabetes [13]. HHcy is also associated with increased risk of neural tube defects [14] and adverse pregnancy outcomes [15]. Every 5 *μ*mol/L increase in Hcy was found to be associated with increased risk of cerebrovascular (59%) and coronary heart diseases (32%) [9].

While Ethiopia has made a remarkable progress in reducing the burden of infectious disease, there is mounting evidence that chronic diseases including cardiovascular diseases (CVDs) and diabetes are on the rise [16]. HHcy could be associated with this rise in the burden on NCDs, but little is known about its prevalence and associated factors. This is unfortunate because such data could help prioritize and inform interventions that aim to reduce the burden of chronic diseases in Ethiopia. Therefore, our study aimed at evaluating the prevalence of HHcy and its associated risk factors in a nationally representative of sample of the Ethiopian adult population. To our knowledge, this is the first nationally representative study that evaluated the prevalence of HHcy and its predictors.

## 2. Methods

### 2.1. Study Design

A nation-wide cross-sectional survey on risk factors for NCDs was conducted (April-June 2015) using the World Health Organization (WHO) STEPwise approach to surveillance (STEPS version 3.1) [17]. The data were collected using the following three steps: Step 1: interview-based assessment of demographic and behavioural characteristics of the study population, Step 2: physical measurements to determine proportion of the study population with raised blood pressure, overweight, and obesity, and Step 3: biochemical measurements to determine the proportion of the study population with diabetes, raised blood glucose, and abnormal lipid profile. In addition to core and expanded modules, some optional modules (khat use) were included in each of the three steps. A total of 35 experienced and well-trained survey teams, each comprising three survey members (1 supervisor and 2 data collectors), were involved in the data collection.

### 2.2. Sampling

A total of 4,175 study participants were included in the national NCD survey. Representative samples of all regions of the country, including Addis Ababa and Dire Dawa city administrations, were collected using the sampling frame of the Ethiopian population and housing census [18]. Study participants meeting the following eligibility criteria were included in the study: 15–69 years of age; permanent residents (>6 months residence) of the survey; and providing consent. Individuals in military camps, dormitories, nursing homes, and refugee camps and pregnant women were excluded from the study. Eligible adults aged 15–69 years in each household were selected using the Kish method [19]. Only one eligible participant per household was selected.

### 2.3. Ethical Approval

Ethical clearance was obtained from the Ethical Review Committees of the Ethiopian Public Health Institute (EPHI) and the National Research and Ethics Review Committee (NERC). Informed consent was obtained from each study participant or from parents/guardians for children less than 18 years of age. The objectives of the study were explained to the participants by the data collectors. The study was conducted according to the principles expressed in the Declaration of Helsinki. Participants with abnormal physical or laboratory findings were counseled and referred to the nearest health facility for further follow-up and medical care.

## 3. Data Collection

### 3.1. Demographic and Lifestyle Factors

Demographic and socioeconomic status, tobacco use, alcohol consumption, fruit and vegetable consumption, physical activity, khat use, and history of raised blood pressure or medication for blood pressure, was assessed.

Physical activity levels were assessed and categorized as high, moderate, and low. High-level physical activity was defined as vigorous-intensity activity at least three days per week achieving at least 1500 metabolic equivalent time (MET) minutes per week, or seven or more days of any combination of walking and moderate- or vigorous-intensity activities achieving at least 3000 MET minutes per week.

Moderate level physical activity was defined as three or more days of vigorous-intensity activity of at least 20 minutes per day; or five or more days of moderate-intensity activity or walking for at least 30 minutes per day; or five or more days of any combination of walking and moderate- or vigorous-intensity activities achieving at least 600 MET minutes per week.

A person not meeting any of the abovementioned criteria was considered to have a low physical activity level [20].

### 3.2. Fruit and Vegetable Consumption

Fruit and vegetable consumption of study participants was captured by asking about the number of days and servings of fruits and vegetables consumed in a typical week. The following serving sizes were considered: for raw green leafy vegetables, 1 serving = one cup; for cooked or chopped vegetables, 1 serving = ½ cup; for fruit (apple, banana, orange, etc.), 1 serving = 1 medium size piece; for chopped, cooked, and canned fruit, 1 serving = ½ cup; and for juice from fruit, 1 serving = ½ cup. Servings were measured by showing pictorial show cards.

### 3.3. Physical Measurements

Height and weight were measured using an equipment that uses infrared and ultrasonic technologies (Deluxe model number GBS-721). The instrument measures body weight and height and calculates body mass index (BMI; kg/m^2^).

Blood pressure measurements were taken three times (in three minutes interval) from the right arm of a sitting participant using a Boso-Medicus Uno instrument (Boso, Germany). The participants were allowed to rest for 15 minutes before measurements were taken. The maximum deviation of cuff pressure measurement was ±3 mmHg, and the three measurements were averaged.

### 3.4. Biochemical Measurements

Both capillary and venous blood samples were obtained after an overnight fast (8–10 h). Capillary blood glucose, total cholesterol, and high-density lipoprotein (HDL) analyses were performed in the field using CardioChek PA Analyzer (Polymer Technology System Inc., Indianapolis, IN). For tHcy analysis, 3–5 ml of whole blood was collected and immediately centrifuged at 3500 rpm to separate serum. Serum samples were transported and stored at −80°C at EPHI-Clinical Chemistry Laboratory until further analyses. Serum tHcy and triglycerides were analyzed using Cobas Integra 400® Plus (Roche Diagnostics GmbH, Mannheim, Germany) at the Clinical Chemistry Laboratory of EPHI [21, 22].

### 3.5. Data Management

Descriptive statistics were calculated for all of the variables. Continuous variables were reported as mean and standard deviation (SD). Categorical variables were reported as frequencies and percentages. Differences in subgroups were analyzed using chi-square. Logistic regression was run to identify risk factors associated with HHcy, after adjusting for covariates gender, age, BMI, residence, marital status, occupational status, level of education, income, physical activity, fruit and/or vegetable consumption, blood glucose, total cholesterol, triglycerides, smoking status, alcohol drinking status, and raised blood pressure or taking blood pressure medication. Hcy values were categorized as normal (Hcy value <15 *μ*mol/L) and HHcy (Hcy value ≥ 15 *μ*mol/L). Odds ratio (OR) and corresponding 95% confidence intervals (CIs) are presented. The data were analyzed using the SPSS software version 22.00 (SPSS Inc. Chicago, IL, USA), and mean differences with *p* values <0.05 were considered statistically significant.

## 4. Results

About 60% of the study participants were females and 39% were 15–29 years of age (Table 1). About half (53%) of the study participants had no formal education. More than two-thirds of the study participants had a high level of physical activity, about 5% were smokers, and >90% consumed alcohol (Table 2). Only 1% met the WHO criteria for adequate fruit and vegetable consumption. About a third of the participants were either undernourished (24%; BMI < 18.5) or overweight/obese (8%; BMI > 25.0). Among study participants, 3% had raised blood sugar, and 17% had high blood pressure or were on blood pressure medication, 24% had high serum triglyceride level, and greater than 60% of the participants had a low HDL level.

**Figure 1:**
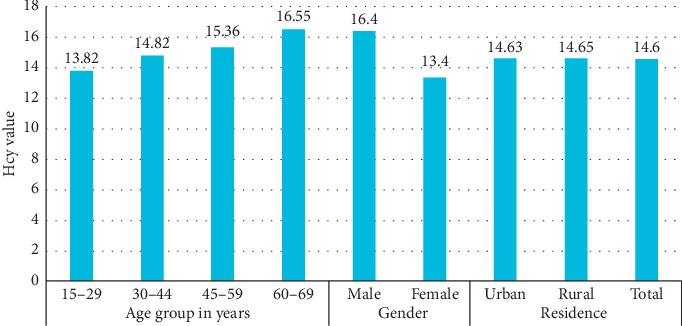
Mean serum level of homocysteine by age, gender, and residence, Ethiopia, 2015.

The mean serum tHcy concentration was 14.6 *μ*mol/L, with 16.4 *μ*mol/L in males and 13.4 *μ*mol/L in females. Overall 38% had HHcy, 49% in males and 30% in females (*p* > 0.05). The prevalence of HHcy increased with age, in both men and women (Table 2). 15–29 years of age had the lowest prevalence of HHcy and 60–69 years of age had the highest prevalence of HHcy (Figure 1).

The results of the logistic regression analyses for HHcy are presented in Table 3. All factors with a *p* value <0.05 in the bivariate analysis were entered into the multivariable model that controlled for confounding factors. In bivariate analysis, we did not find any association between HHcy and BMI (*p*=0.508), marital status (*p*=0.203), total cholesterol level (*p*=0.234), triglyceride level (*p*=0.065), and current alcohol intake (*p*=0.093). Logistic regression analysis for HHcy using age group, gender, regions, occupational status, level of education, quartiles of income, fruit and/or vegetable consumption, blood glucose level, HDL, smoking status, and raised blood pressure or taking medication as covariates was performed.

Being male (AOR = 2.16; 95% CI, 1.79–2.62), increased age (AOR = 3.19; 95% CI, 1.90, 5.34), residing in Somali or Dire Dawa region (AOR = 3.37; 95% CI, 1.46–7.78), raised blood pressure (AOR = 1.55; 1.27–1.91), and low fruit and vegetable consumption relative to WHO recommendations (AOR = 9.57; 2.83, 32.30) were independent predictors of increased HHcy.

## 5. Discussion

The present study has identified that the prevalence of HHcy is alarmingly high. Age, male gender, low fruit and vegetable consumption, and high blood pressure or taking blood pressure medication were the major risk factors. There was also high regional variability in the prevalence of HHcy, with an increased risk of HHcy in some regions (e.g., Dire Dawa and Somali).

The high prevalence of HHcy in the Ethiopian population is concerning and could in part explain the increased mortality and morbidity from NCDs in Ethiopia. Indeed, Misganaw et al. [16] have recently shown that ischemic heart disease, ischemic stroke, and diabetes are ranked 1^st^, 6^th^, and 8^th^ in the cause of age-standardized death for Ethiopia. While several lifestyle factors such as physical inactivity and smoking are often found to be associated with HHcy [23], these were not significant predictors of HHcy in this Ethiopian sample. This could be due to the high to medium level of physical activity observed in most of our study participants and the relatively low level of smoking.

The current study showed that HHcy was higher in males, which is consistent with previous studies [24, 25]. The gender difference in HHcy is likely to be associated with sex-related hormones, the higher phosphocreatine synthesis in males, and the higher rate of remethylation and transmethylation in females than in males [26]. In this study, similar to previous findings [25, 27], the prevalence of HHcy increased with age. This might be due to reduced bioavailability of folate with age [28].

Low consumption of fruits and vegetables and high blood pressure were major predictors of HHcy. Indeed, many studies have documented the advantage of increased fruit and vegetable consumption in promoting health and preventing NCDs including cardiovascular diseases [29]. There could be several explanations for this: First, dark green leafy vegetables are particularly good sources of folate, and adequate folate status could decrease the risk of HHcy. The low level of fruit and vegetable consumption is in line with the findings of the National Food Consumption Survey and is further supported by the relatively high prevalence of folate deficiency in Ethiopia [30, 31].

Second, fruits and vegetables are good sources of antioxidants and polyphenols that can help lower total serum Hcy, prevent lipid oxidation in arterial vessel walls, lower blood pressure, and improve endothelial function. This is supported by a recent study that used a quantile regression approach to show the inverse relationship between fruit and vegetable consumption and serum Hcy [32].

The high regional variability in the prevalence of HHcy could thus partly be attributed to variability in regional dietary patterns, but this would warrant further studies. For instance, Somali and Dire Dawa had the highest HHcy prevalence. These regions also have one of the lowest consumption of fruits and vegetables [30, 33]. Different studies indicated that Hcy is considered as a risk factor for hypertension even though the causal relationship between hypertension and Hcy is limited. HHcy is often accompanied by increased reactive oxygen species, matrix metalloproteinase, and decreased endothelial nitric oxide which all causes stiffness and vascular constriction that can lead to hypertension [34]. In this study, raised blood pressure or taking blood pressure medication was associated with HHcy.

The present study had several strengths, including the nationally representative sampling, large sample size, investigation of lifestyle factors, anthropometry, and several biomarkers associated with HHcy. However, certain limitations need to be considered when interpreting our findings. First, the present study is cross-sectional, and hence, causal inferences cannot be made. Second, even though MTHFR 677CT polymorphism and intake of folate and vitamin B12 can all affect serum tHcy concentrations, they were not assessed in the present study; however, fruit and vegetable consumption was assessed to reflect dietary patterns.

## 6. Conclusions

Despite the abovementioned limitations, the present study is the first nationally representative study that determined the prevalence of HHcy and its associated factors. The study highlighted the alarmingly high prevalence of HHcy, but also showed that increased age, being male, increased blood pressure or taking blood pressure medication, and low fruit and vegetable consumption were key predictors of HHcy. Improving diets through the promotion of fruits and vegetable consumption could be an effective strategy to prevent the adverse effects (i.e., CVD) associated with HHcy.

## Figures and Tables

**Table 1:** Sociodemographic characteristics of the study participants, Ethiopia, 2015.

Variables *n* (%)	Total	Male	Female
	4175 (100)	1722 (41.2)	2453 (58.8)
Age (years)			
15–29	1625 (38.9)	626 (36.4)	999 (40.7)
30–44	1526 (36.6)	621 (36.1)	905 (36.9)
45–59	739 (17.7)	337 (19.6)	402 (16.4)
60–69	285 (6.8)	138 (8.0)	147 (6.0)

Residence			
Urban	952 (22.8)	283 (16.4)	669 (27.3)
Rural	3223 (77.2)	1439 (83.6)	1784 (72.7)

BMI (kg/m^2^)			
Low (≤18.5)	956 (23.9)	452 (26.4)	504 (22.0)
Normal (19–24)	2703 (67.5)	1161 (67.9)	1542 (67.2)
High (≥25)	348 (8.7)	98 (5.7)	250 (10.9)

Geographic region			
Tigray	682 (16.3)	257 (14.9)	425 (17.3)
Afar	167 (4.0)	63 (3.7)	104 (167)
Amhara	794 (19.0)	343 (19.9)	451 (18.4)
Oromiya	961 (23.0)	392 (22.8)	569 (23.2)
Somali	276 (6.6)	105 (6.1)	171 (7.0)
B. Gumuz	214 (5.1)	104 (6.0)	110 (4.5)
SNNPR	527 (12.6)	232 (13.5)	295 (12.0)
Gambela	239 (5.7)	131 (7.6)	108 (4.4)
Harari	59 (1.4)	22 (1.3)	37 (1.5)
Dire Dawa	51 (1.2)	30 (1.7)	21 (0.9)
Addis Ababa	205 (4.9)	43 (2.5)	162 (6.6)

Marital status			
Single	1326 (31.8)	466 (27.1)	860 (35.1)
Married or cohabiting	2848 (68.2)	1256 (72.9)	1592 (64.9)

Occupational status			
Employed	379 (9.2)	216 (12.8)	163 (6.7)
Skilled worker	2336 (56.7)	1248 (74.2)	1088 (44.6)
Housewife	959 (23.3)	8 (0.5)	951 (39.0)
Unemployed	446 (10.8)	211 (12.5)	235 (446)

Level of education			
No formal education	2227 (53.3)	712 (41.3)	1515 (61.8)
Primary education	1472 (35.3)	762 (44.3)	710 (28.9)
Secondary education	259 (6.2)	113 (6.6)	146 (6.0)
College and above	217 (5.2)	135 (7.8)	82 (3.3)

Income (x 1000 birr)			
<12	2335 (68.1)	922 (63.3)	1413 (71.6)
12–18	422 (12.3)	200 (13.7)	222 (11.2)
18–23	206 (6.0)	115 (7.9)	91 (4.6)
23–30	213 (6.2)	96 (6.6)	117 (5.9)
>30	254 (7.4)	123 (8.4)	131 (6.6)

BMI, = body mass index; low BMI ≤ 18.5 kg/m^2^; normal BMI = 18.5–24.99 kg/m^2^; high BMI ≥ 25 kg/m^2^.

**Table 2:** Lifestyle factors and biochemical measures of study participants, Ethiopia, 2015.

Variables *n* (%)	Total	Male	Female	*p* value
	4175 (100)	1722 (41.2)	2453 (58.8)	
Physical activity level				
Low	609 (14.7)	159 (9.3)	450 (18.5)	**<0.001**
Moderate	679 (16.4)	178 (10.4)	501 (20.6)	
High	2850 (68.9)	1372 (80.3)	1478 (60.8)	

Current smoking				
Yes	233 (5.6)	202 (11.7)	31 (1.3)	**<0.001**
No	3942 (94.4)	1520 (88.3)	2422 (98.7)	

Current alcohol intake				
Yes	1708 (92.8)	833 (95.3)	875 (90.6)	**<0.001**
No	132 (7.2)	41 (4.7)	91 (9.4)	

Fruit and vegetable consumption				
Yes	50 (1.2)	18 (1.0)	32 (1.3)	0.448
No	4125 (98.8)	1704 (99.0)	2421 (98.7)	

Blood sugar level				
Normal (<110 mg/dl)	3740 (92.5)	1546 (92.4)	2194 (92.5)	0.140
Impaired fasting glucose (110–125 mg/dl)	185 (4.6)	69 (4.1)	116 (4.9)	
Raised blood sugar (≥126 mg/dl)	119 (2.9)	58 (3.5)	61 (2.6)	

Raised blood pressure/on blood pressure medication^Δ^				
Yes	722 (17.3)	280 (16.3)	442 (18.0)	0.139
No	3453 (82.7)	1442 (83.7)	2011 (82.0)	

Total cholesterol level (mg/dl)				
<200	3796 (92.6)	1621 (95.3)	2175 (90.7)	**<0.001**
≥200	304 (7.4)	80 (4.7)	224 (9.3)	

Triglyceride level (mg/dl)				
<150	3192 (76.5)	1319 (76.6)	1873 (76.4)	0.856
≥150	983 (23.5)	403 (23.4)	580 (23.6)	

HDL level ^*Φ*^				
Normal	1400 (34.7)	673 (40.5)	1400 (30.7)	**<0.001**
Low	2630 (65.3)	990 (59.5)	2630 (69.3)	

Homocysteine (*μ*mol/L)^b^	14.6 ± 6.8	16.4 ± 7.5	13.4 ± 6.0	**<0.001**

Homocysteine level (*μ*mol/L)				
<15	2593 (62.1)	879 (51.0)	1714 (69.9)	**<0.001**
≥15	1582 (37.9)	843 (49.0)	739 (30.1)	

^Δ^SBP = systolic blood pressure; DBP = diastolic blood pressure; raised blood pressure (SBP ≥ 140 and/or DBP ≥ 90 mmHg). ^*Φ*^HDL level: low HDL level for males, <40 mg/dl, and for females, <50 mg/dl. ^b^Values are mean ± standard deviation.

**Table 3:** Factors associated with hyperhomocysteinaemia in the Ethiopian adult population, 2015.

Variables *n* (%)	Homocysteine concentration (Hcy)		Adjusted OR (95% CI)	*p* value
	<15 *μ*mol/L	≥15 *μ*mol/L		
Sex				
Female	1714 (69.9)	739 (30.1)	1	**<0.001**
Male	879 (51.0)	843 (49.0)	2.16 (1.79, 2.62)	

Age (years)				
15–29	1107 (68.1)	518 (31.9)	1	
30–44	934 (61.2)	592 (38.8)	1.31 (1.08, 1.58)	**0.007**
45–59	412 (55.8)	327 (44.2)	1.80 (1.41, 2.30)	**<0.001**
60–69	140 (49.1)	145 (50.9)	2.06 (1.47, 2.90)	**<0.001**

Geographic region				
Addis Ababa	109 (53.2)	96 (46.8)	1	
Tigray	467 (68.5)	215 (31.5)	0.50 (0.33, 0.76)	**0.001**
Afar	85 (50.9)	82 (49.1)	1.23 (0.74, 2.03)	0.433
Amhara	595 (74.9)	199 (25.1)	0.34 (0.22, 0.51)	**<0.001**
Oromia	639 (66.5)	322 (33.5)	0.53 (0.36, 0.79)	**0.002**
Somali	84 (30.4)	192 (69.6)	3.19 (1.90, 5.34)	**<0.001**
B. Gumuz	103 (48.1)	111 (51.9)	1.02 (0.62, 1.67)	0.954
SNNPR	359 (68.1)	168 (31.9)	0.49 (0.32, 0.74)	**0.001**
Gambela	105 (43.9)	134 (56.1)	1.47 (0.82, 2.62)	0.194
Harari	33 (55.9)	26 (44.1)	1.31 (0.56, 3.07)	0.536
Dire Dawa	14 (275)	37 (72.5)	3.37 (1.46, 7.78)	**0.005**

Occupational status				
Unemployed	281 (63.0)	165 (37.0)	1	
Employed	209 (55.1)	170 (44.9)	1.17 (0.76, 1.82)	0.470
Skilled worker	1437 (61.5)	899 (38.5)	1.11 (0.77, 1.59)	0.583
Housewife/homemaker	646 (67.4)	313 (32.6)	1.04 (0.69, 1.55)	0.863

Level of education				
No formal education	1365 (52.6)	862 (54.5)	1	
Primary education	960 (37.0)	512 (32.4)	0.96 (0.79, 1.17)	0.697
Secondary education	157 (6.1)	102 (6.4)	1.18 (0.82, 1.70)	0.379
College and above	111 (4.3)	106 (6.7)	1.52 (0.98, 2.37)	0.063

Income quartiles (×1000 birr)				
<12	1491 (69.6)	844 (65.6)	1	
12–18	257 (12.0)	165 (12.8)	1.17 (0.85, 1.62)	0.337
18–23.3	110 (5.1)	96 (7.5)	1.09 (0.75, 1.59)	0.643
23.3–30	128 (6.0)	85 (6.6)	1.22 (0.79, 1.89)	0.380
>30	157 (7.3)	97 (7.5)	1.16 (0.76, 1.78)	0.493

Physical activity level				
Low	385 (15.0)	224 (14.3)	1	
Moderate	454 (17.6)	225 (14.4)	1.02 (0.77, 1.36)	0.889
High	1734 (67.4)	1116 (71.3)	1.30 (1.02, 1.66)	0.034

Fruit and vegetable consumption				
Yes	46 (1.8)	4 (0.3)	1	
No	2547 (98.2)	1578 (99.7)	9.57 (2.83, 32.30)	**<0.001**

Blood sugar level (mg/dl)				
Normal (<110)	2364 (63.2)	1376 (36.8)	1	
Impaired fasting glucose (110–125)	95 (51.4)	90 (48.6)	1.26 (0.73, 2.16)	0.413
Raised blood sugar (≥126)	71 (59.7)	48 (40.3)	1.44 (0.76, 2.74)	0.266

High-density lipoprotein level (HDL)^*Φ*^				
Normal	824 (58.9)	576 (41.1)	1	
Low	1691 (64.3)	939 (35.7)	1.09 (0.93, 1.29)	0.291

Currently smoking				
No	2487 (63.1)	1455 (36.9)	1	
Yes	106 (45.5)	127 (54.5)	0.84 (0.58, 1.20)	0.336

Raised blood pressure/on blood pressure medication Δ				
No	2193 (63.5)	1260 (36.5)	1	
Yes	400 (55.4)	322 (44.6)	1.55 (1.27, 1.91)	**<0.001**

^*Φ*^HDL level: low HDL level for males, <40 mg/dl, and for females, <50 mg/dl. ^Δ^SBP = systolic blood pressure; DBP = diastolic blood pressure; raised blood pressure (SBP ≥ 140 and/or DBP ≥ 90 mmHg).

## Data Availability

The datasets can be made available from the corresponding author upon reasonable request.
